# Development and Characterization of Carrageenan-Based Hydrogels Enriched with *Calendula officinalis* Extract for Topical Skin Applications

**DOI:** 10.3390/polym18192436

**Published:** 2026-10-07

**Authors:** Justyna Kozlowska, Anna Bakula, Katarzyna Noryca

**Affiliations:** 1 Faculty of Chemistry, Nicolaus Copernicus University in Torun, Gagarina 7, 87-100 Torun, Poland; lipinska2501@gmail.com (A.B.); knoryca6@gmail.com (K.N.); 2 Institute of Advanced Studies, Nicolaus Copernicus University in Torun, Wilenska 4, 87-100 Torun, Poland

**Keywords:** carrageenan, microspheres, gelatin, encapsulation, hydrogels, *Calendula officinalis* L., cosmetic applications

## Abstract

Carrageenan-based hydrogels are promising matrices for topical cosmetic applications because their highly hydrated polymer networks can be modified to incorporate bioactive compounds and adjust functional properties. In this study, *Calendula officinalis* flower extracts were incorporated into κ-carrageenan-based hydrogels either directly or after loading into chemically crosslinked gelatin microspheres. The obtained formulations were characterized in terms of morphology, compressive modulus, swelling behaviour, thermal stability, and selected biophysical skin parameters. The hydrogels showed formulation-dependent differences in surface morphology, mechanical properties, water uptake, and thermal behaviour. The compressive modulus ranged from 14.10 ± 1.15 to 23.20 ± 1.46 kPa, with the highest value observed for the hydrogel containing directly incorporated hydroglycolic extract. This formulation also exhibited the lowest degree of swelling, whereas higher swelling was generally observed in microsphere-containing systems. All formulations showed three main stages of thermal mass loss. Preliminary skin measurements showed no pronounced persistent increase in transepidermal water loss or marked changes in the A* parameter following hydrogel application. The results show that direct and microsphere-mediated incorporation represent distinct formulation strategies resulting in different physicochemical properties of carrageenan-based hydrogels. Preliminary skin measurements additionally provided initial information on their short-term response following topical application.

## 1. Introduction

Hydrogels have gained increasing interest as cosmetic carriers owing to their ability to accommodate bioactive compounds within highly hydrated polymeric networks. The high water content of these systems enables intimate contact with the skin surface and contributes to the formation of a temporary occlusive film, which may improve *stratum corneum* hydration and support active ingredient delivery [1]. Compared with conventional creams and emulsions, hydrogel masks allow easier modulation of physicochemical characteristics, including swelling behaviour, mechanical properties, and release performance, by varying polymer composition and network organization [2].

Carrageenans are widely used marine polysaccharides for hydrogel preparation. These sulfated galactans, isolated from red algae, are classified into κ-, ι-, and λ-carrageenan according to the content and distribution of sulfate groups within the polymer chain [3]. Due to their biodegradability, biocompatibility, and low toxicity, carrageenans have been widely investigated for food, food packaging, cosmetic, and biomedical applications [4,5,6,7]. Carrageenans are characterized by high water-holding capacity and function as thickening, gelling, and stabilizing agents in a variety of formulations. In particular, κ-carrageenan undergoes cation-mediated gelation, predominantly in the presence of potassium and calcium ions, resulting in thermoreversible hydrogel networks with adjustable mechanical properties [3,7,8].

Carrageenan-based hydrogels have also been investigated for topical cosmetic applications, including hydrogel masks and patches. Their high water content, gel-forming ability, and compatibility with various active ingredients make them suitable matrices for the development of skin-contact formulations [6,9].

The physicochemical and biological properties of carrageenan-based hydrogels can be further tailored by modifying their polymer composition. Carrageenan is frequently blended with other natural polymers, such as chitosan [10], sodium alginate and starch [11], or synthetic polymers, such as poly(vinyl alcohol) [12] and polyvinylpyrrolidone [13], to modify mechanical stability, swelling behaviour, degradation profile, and cellular response. Such systems have been developed for drug delivery, tissue engineering, and wound healing applications.

In addition to modifying polymer composition, bioactive compounds have been incorporated into carrageenan-based hydrogels to confer specific biological properties [4,6,14,15]. For example, incorporation of R-phycoerythrin into κ-carrageenan hydrogel enhanced its antioxidant, antibacterial, and wound-healing activities [16]. Moreover, silver nanoparticles have been incorporated into carrageenan-based matrices to impart antimicrobial properties and enhance their suitability for wound-dressing applications [13].

Particulate carriers have been widely investigated as delivery systems for bioactive compounds because they may improve compound stability and facilitate their incorporation into polymeric matrices [17,18,19,20]. Among them, gelatin microspheres are particularly attractive owing to their biocompatibility, biodegradability, and ability to absorb hydrophilic compounds [21,22,23,24]. Following incorporation into hydrogel systems, they may also affect the internal organization and physicochemical properties of the polymer network. However, their application as carriers of plant-derived extracts in carrageenan-based cosmetic hydrogels has received little attention. Therefore, in this study, pot marigold flower extract was incorporated into κ-carrageenan hydrogels either directly into the polymer matrix or after prior loading into chemically crosslinked gelatin microspheres.

Pot marigold (*Calendula officinalis* L.) is one of the most widely used medicinal plants in topical formulations owing to its anti-inflammatory, antioxidant, and regenerative properties. It is an annual plant species in the family Asteraceae [25]. The main components of marigolds are steroids, terpenoids, triterpenoids, flavonoids, phenolic acids, and carotenes. Also, rutin, caffeic acid, and chlorogenic acid were isolated from this plant [26]. Rutin, one of the main flavonoids present in many plant materials, is responsible for anti-inflammatory, antibacterial, and hepatoprotective effects [27]. Caffeic acid and chlorogenic acid present in pot marigold flowers are commonly associated with antioxidant effects [28]. Consequently, *Calendula officinalis* flower extracts have been used in cosmetic and dermatological preparations for irritated, sensitive, and damaged skin.

The stability and distribution of plant-derived bioactive compounds within polymeric systems can be improved by their incorporation into particulate carriers prior to introduction into the hydrogel matrix. Gelatin microspheres represent suitable carriers owing to their biocompatibility, biodegradability, high swelling capacity, and ability to absorb hydrophilic compounds [29,30]. Following incorporation into hydrogels, they may also affect the internal architecture and physicochemical characteristics of the resulting materials.

The present study was designed to compare two formulation approaches for incorporating *Calendula officinalis* flower extracts into κ-carrageenan-based cosmetic hydrogels: direct incorporation into the carrageenan matrix and incorporation via extract-loaded gelatin microspheres. The obtained materials were characterized with respect to their morphology, swelling behaviour, thermal stability, mechanical properties, and selected skin-related parameters.

## 2. Materials and Methods

### 2.1. Materials

Collagen type I was obtained in our laboratory from fish scales of *Esox lucius* [31]. κ-carrageenan, Span^®^85, gelatin type A, 1-ethyl-3(3-dimethylaminopropyl) carbodiimide (EDC), N-hydroxysuccinimide (NHS), tris(hydroxymethyl)aminomethane hydrochloride (Tris-HCl), and sorbitol were purchased from Sigma-Aldrich (Poznan, Poland). Hydrochloric acid, acetic acid, sodium acetate anhydrous, and sodium chloride were obtained from Avantor Performance Materials Poland S.A. (Gliwice, Poland). Ethyl alcohol, glycerin, paraffin oil, and disodium edetate dihydrate were supplied by Stanlab (Lublin, Poland). Calcium chloride dihydrate and propylene glycol were purchased from Chempur (Piekary Śląskie, Poland). The hydroglycolic *Calendula officinalis* flower extract (CALENDULA-EXTRACT H.G. 42300) was acquired from Provital S.A. (Barcelona, Spain). According to the manufacturer’s specification, it was an extract of *Calendula officinalis* L. flowers in a propylene glycol/water medium (80:20), with a plant-to-extract ratio of 1:2 and a dry residue of at least 2.5%. A dry *Calendula officinalis* extract was purchased from Zrob Sobie Krem (Prochowice, Poland). According to the supplier’s certificate of analysis, the powdered extract contained 5% lutein; the maltodextrin, moisture, and ash contents were 9.03%, 2.85%, and 3.00%, respectively.

### 2.2. Preparation of Gelatin Microspheres

Gelatin microspheres (GEL) were prepared using a water-in-oil (W/O) emulsification method. Briefly, 5 mL of a 20% (*w/v*) aqueous gelatin solution (type A porcine skin gelatin) was prepared by dissolving the polymer at 40 °C for 30 min. Subsequently, 40 mL of liquid paraffin containing 1% (*w/v*) Span^®^85 was added, and the mixture was homogenized at 3000 rpm for 20 min using an IKA T25 digital ULTRA-TURRAX homogenizer to obtain a stable W/O emulsion. The gelatin droplets were then chemically crosslinked with 50 mM EDC and 25 mM NHS for 4 h at room temperature. Following crosslinking, the microspheres were collected by centrifugation (10,000 rpm, 10 min; Eppendorf 5805R) and washed four times with 96% ethanol to remove residual reagents and oil. Finally, the microspheres were frozen at −20 °C and lyophilized at −55 °C under a pressure of 5 Pa for 24 h. The preparation protocol was adapted from our previously published methodology [24].

### 2.3. Isolation of Collagen from Esox lucius

Collagen was extracted from the scales of common pike (*Esox lucius*) collected from Lake Dejguny using a previously reported procedure developed by Kozłowska et al. [31]. The isolation protocol consisted of several sequential steps, including scale cleaning, demineralization, collagen extraction with acetic acid, salt-induced precipitation, centrifugation, dialysis, freezing, and subsequent freeze-drying to obtain purified collagen.

### 2.4. Formulation of Hydrogel Matrices

Five κ-carrageenan-based hydrogel (CARR) formulations were prepared to compare two formulation approaches for incorporating *Calendula officinalis* preparations: direct addition to the polymer solution and incorporation via extract-loaded gelatin microspheres. All formulations contained the same concentrations of κ-carrageenan (2%, *w/v*), sorbitol (20% of a 60% solution), glycerin (1.5%, *v/v*), propylene glycol (1%, *v/v*), and fish collagen (0.1%, *w/v*). The formulations differed in the type of *Calendula officinalis* preparation used and in the incorporation approach, i.e., either direct addition to the polymer solution or incorporation via extract-loaded gelatin microspheres. Extract-loaded gelatin microspheres were added to the corresponding hydrogel formulations at a concentration of 1% (*w/v*). The composition of all hydrogel formulations is presented in Table 1.

Before hydrogel preparation, a 60% (*w/v*) sorbitol solution and a 0.5 M calcium chloride solution were prepared. Aqueous solutions of the two *Calendula officinalis* preparations were prepared on the basis of their dry-matter content. For direct incorporation into the hydrogel formulations, the solutions were adjusted to provide a nominal dry-matter concentration of 0.1% (*w/v*). For the hydroglycolic extract, the required amount of the commercial preparation was calculated assuming the minimum manufacturer-specified dry residue of 2.5%. The dry *Calendula officinalis* extract was dissolved directly in water to obtain a 0.1% (*w/v*) solution.

For microsphere loading, aqueous extract solutions corresponding to a nominal dry-matter concentration of 2% (*w/v*) were prepared analogously. The hydroglycolic preparation was diluted based on its declared dry-residue content, whereas the dry extract was dissolved directly in water. Gelatin microspheres were immersed in the respective 2% (*w/v*) extract solutions at room temperature for 24 h.

**Figure 1:**
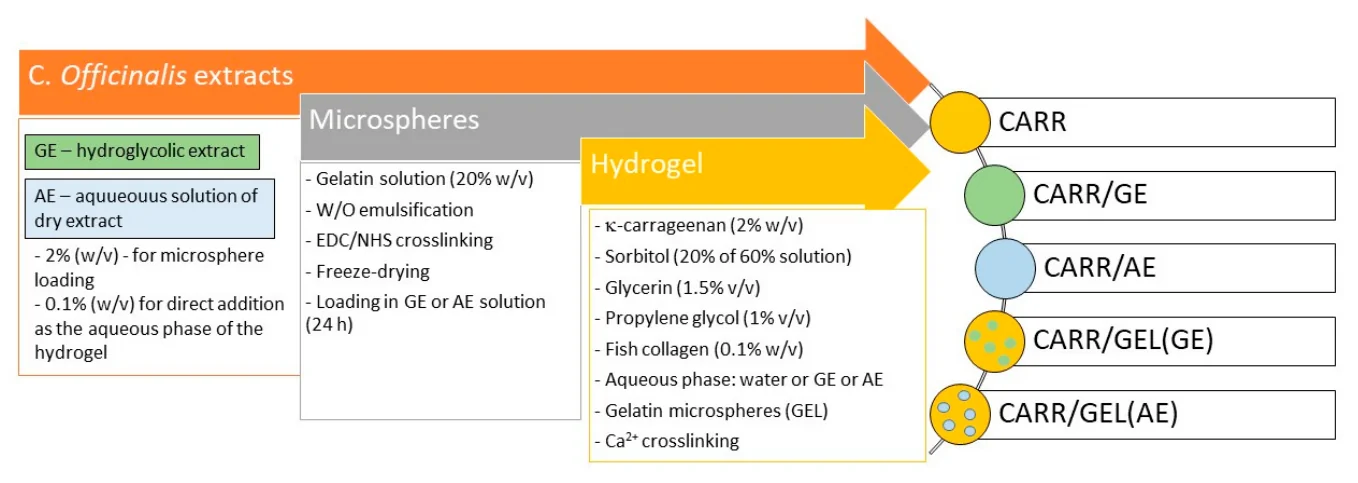
Schematic representation of the preparation procedure for the different κ-carrageenan-based hydrogel formulations.

For hydrogel fabrication, the required amounts of κ-carrageenan, sorbitol solution, glycerin, propylene glycol, collagen, and the appropriate extract solution or extract-loaded microspheres were mixed and heated in a water bath until the polymer components were fully dissolved. Formulations containing gelatin microspheres were additionally homogenized using an IKA T25 digital ULTRA-TURRAX homogenizer to ensure their uniform distribution throughout the polymer matrix. The resulting mixtures were cast into Petri dishes and ionically crosslinked by immersion in 0.5 M CaCl_2_ solution for 5 min. Finally, the hydrogels were stored at −20 °C until further characterization. The procedure for preparing the individual types of carrageenan-based hydrogels is presented in Figure 1.

### 2.5. Characteristics of Test Methods

#### 2.5.1. Imaging of Materials

The morphology of the obtained materials was evaluated using photographic imaging, optical microscopy, and scanning electron microscopy (SEM). Macroscopic photographs of the hydrogel matrices were taken immediately after preparation and following freeze-drying using a Samsung Galaxy J4+ and Motic SMZ-171 BLED stereomicroscope. SEM analysis was performed using a Quanta 3D FEG scanning electron microscope (Quorum Technologies, Cambridge, UK) to examine the surface morphology of the crosslinked microspheres and freeze-dried hydrogel matrices. Before SEM observation, the samples were sputter-coated with a thin layer of gold and palladium.

#### 2.5.2. Mechanical Properties

The compressive mechanical properties of the hydrogel matrices were evaluated using a universal testing machine (Z0.5, Zwick/Roell, Ulm, Germany) equipped with a 0.5 kN load cell. Cylindrical specimens (approximately 16 mm in diameter and 21 mm in height) were prepared by casting the hydrogel formulations into cylindrical polystyrene molds. Before testing, the dimensions of each specimen were measured. The samples were placed between two compression plates and compressed at a crosshead speed of 2 mm min^−1^ until reaching 10% strain. The measurements were performed at room temperature using the testXpert II software (Zwick/Roell, Ulm, Germany). The compressive modulus (*E*_c_) was determined from the slope of the linear region of the stress–strain curve corresponding to 2–5% strain. All measurements were performed in quintuplicate, and the results are presented as mean ± standard deviation.

#### 2.5.3. Swelling Test

The swelling behaviour of the hydrogel matrices was evaluated gravimetrically. Cylindrical hydrogel specimens of identical dimensions were weighed to determine their initial mass (W_0_) and subsequently immersed in acetate buffer (pH 5.4) at room temperature. At predetermined time intervals (0.25, 1, 2, 3, 4, 6, and 24 h), the samples were removed from the medium, gently blotted with filter paper to remove excess surface water, and immediately weighed (W_t_). The swelling degree (SD) was calculated according to Equation (1). All measurements were performed in triplicate, and the results are presented as mean ± standard deviation.

(1) $$\mathrm{SD}\left(\%\right)=\left[\frac{{(W}_{t}-W_{0})}{W_{0}}\right]\times 100$$

#### 2.5.4. Thermal Stability

Thermogravimetric analysis (TGA) was performed using a TA Instruments SDT 2960 Simultaneous TGA-DTA (New Castle, DE, USA) under a nitrogen atmosphere at a heating rate of 10 °C/min over the temperature range of 25–600 °C.

#### 2.5.5. Preliminary Assessment of Skin Parameters

Selected biophysical skin parameters were evaluated following short-term application of the prepared hydrogel matrices using a Multi Probe Adapter MPA 5 (Courage + Khazaka Electronic GmbH, Cologne, Germany). Transepidermal water loss (TEWL) and skin colour were determined using a Tewameter^®^ TM 300 and Skin-Colorimeter CL 400, respectively.

The pilot study was performed on three healthy female volunteers (22–23 years old) with comparable skin characteristics. The TEWL values measured in all subjects indicated well-preserved skin barrier function. Furthermore, all volunteers exhibited a very light skin tone, as reflected by ITA values Before the measurements, the volunteers were allowed to acclimatize to the laboratory conditions. Individual hydrogel formulations were applied to separate 4 × 4 cm areas marked on the volar forearm, while an untreated skin area served as the control. The hydrogels remained in contact with the skin for 20 min, after which they were removed. Measurements were carried out before hydrogel application (baseline), immediately after hydrogel removal, and at 10, 30, 60, 120, and 180 min after removal. All measurements were performed under controlled environmental conditions (20–22 °C, relative humidity 40–60%).

TEWL was determined by comparing the treated and untreated (control) skin areas at each measurement time point. TEWL and skin colour measurements were carried out in triplicate. The results are presented as mean ± standard deviation.

### 2.6. Statistical Analysis

Statistical analysis of the mechanical data was performed using SigmaPlot 14.0 software (Systat Software, San Jose, CA, USA). Data normality was assessed using the Shapiro–Wilk test. Differences between the hydrogel formulations were evaluated using one-way analysis of variance (ANOVA), followed by Dunnett’s post hoc test for multiple comparisons with the control CARR formulation. Statistical significance was established at *p* < 0.05. Due to the pilot nature of the skin study and the small number of volunteers (*n* = 3), the skin measurements were analysed descriptively and are presented as mean ± standard deviation.

## 3. Results

### 3.1. Imaging of Materials

**Figure 2:**
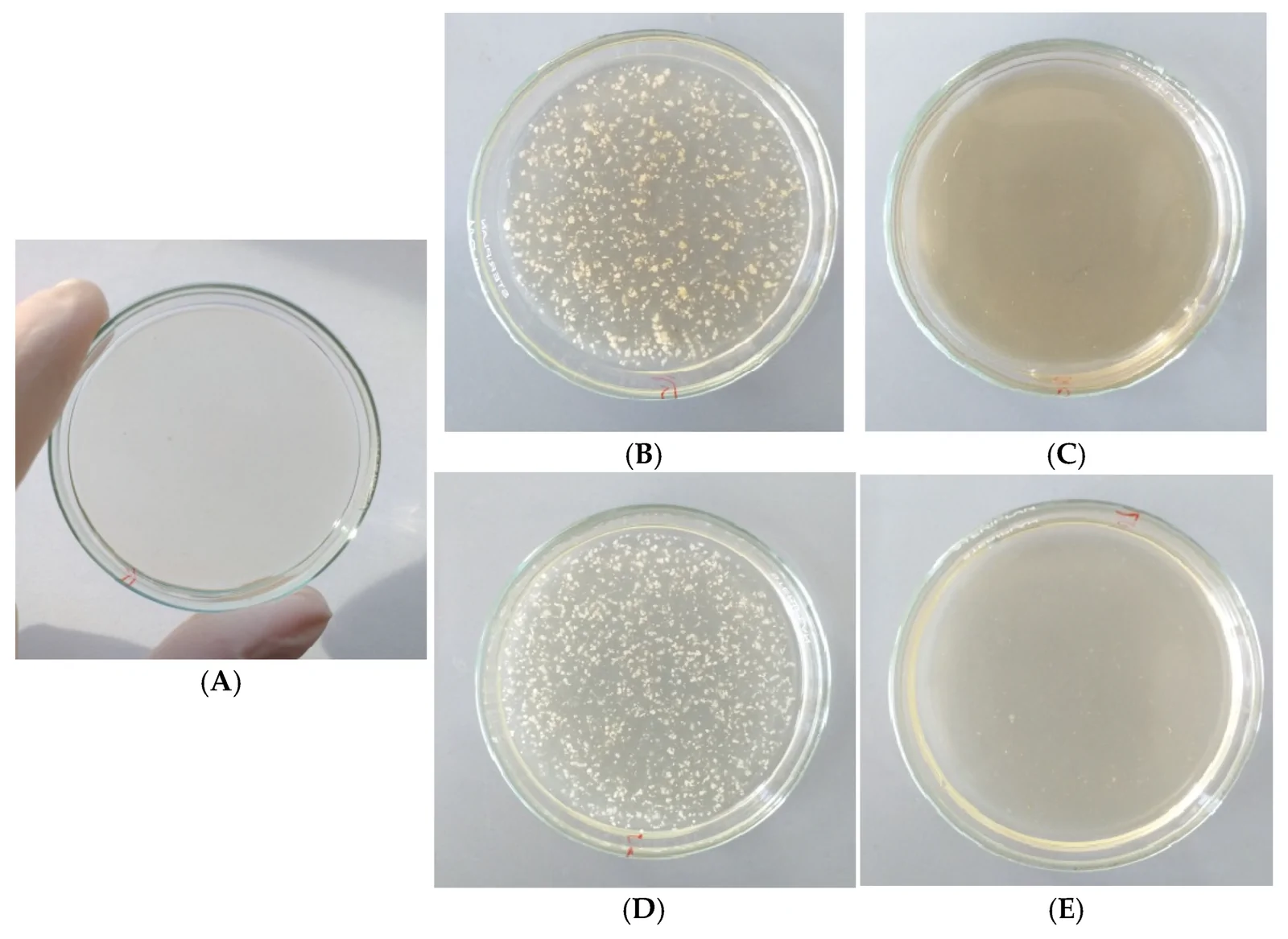
Digital camera imaging of matrices: (**A**)—without extract and microspheres (CARR), (**B**)—with a hydroglycolic extract incorporated in gelatin microspheres (CARR/GEL (GE)), (**C**)—with a hydroglycolic extract in the entire volume of the matrix (CARR/GE), (**D**)—with an aqueous solution of dry extract incorporated in gelatin microspheres (CARR/GEL (AE)), (**E**)—with an aqueous solution of dry extract in the entire volume of the matrix (CARR/AE).

Figure 2 shows photographs of carrageenan-based hydrogels immediately after their preparation. Macroscopic examination of the freshly prepared hydrogels showed that all formulations formed continuous and homogeneous matrices. The control CARR hydrogel was transparent, whereas the incorporation of *Calendula officinalis* extracts resulted in a slight change in colour and transparency. In the formulations containing extract-loaded gelatin microspheres, the dispersed microspheres were clearly visible throughout the hydrogel matrix.

**Figure 3:**
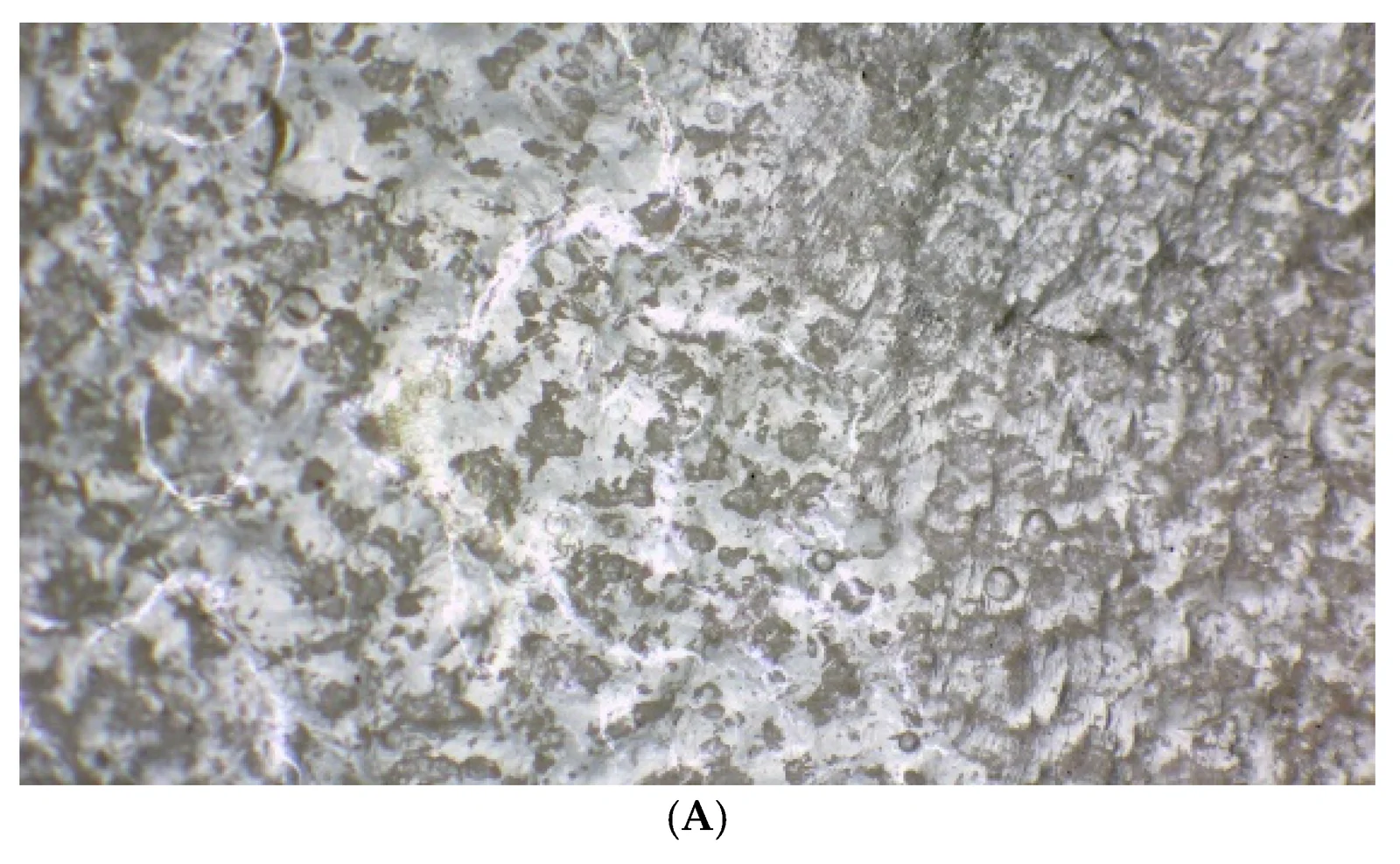

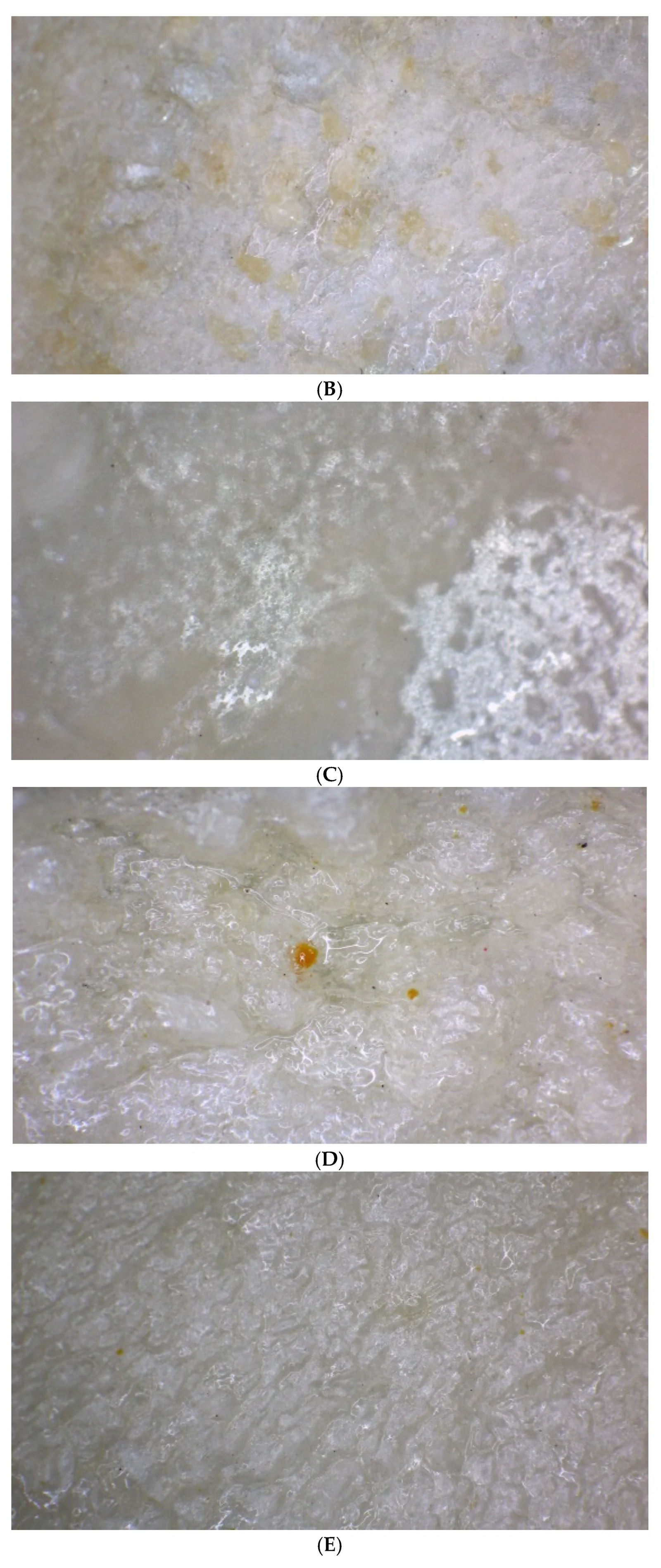
Stereomicroscopic images of lyophilized matrices: (**A**)—without extract and microspheres (CARR), (**B**)—with a hydroglycolic extract incorporated in gelatin microspheres (CARR/GEL (GE)), (**C**)—with a hydroglycolic extract in the entire volume of the matrix (CARR/GE), (**D**)—with an aqueous solution of dry extract incorporated in gelatin microspheres (CARR/GEL (AE)), (**E**)—with an aqueous solution of dry extract in the entire volume of the matrix (CARR/AE).

Stereomicroscopic images of the freeze-dried hydrogel matrices are presented in Figure 3. The control CARR matrix exhibited a porous, irregular structure, whereas the extract-containing formulations showed more compact, heterogeneous surfaces. Differences were also visible between hydrogels containing the extracts directly and those containing extract-loaded gelatin microspheres.

**Figure 4:**
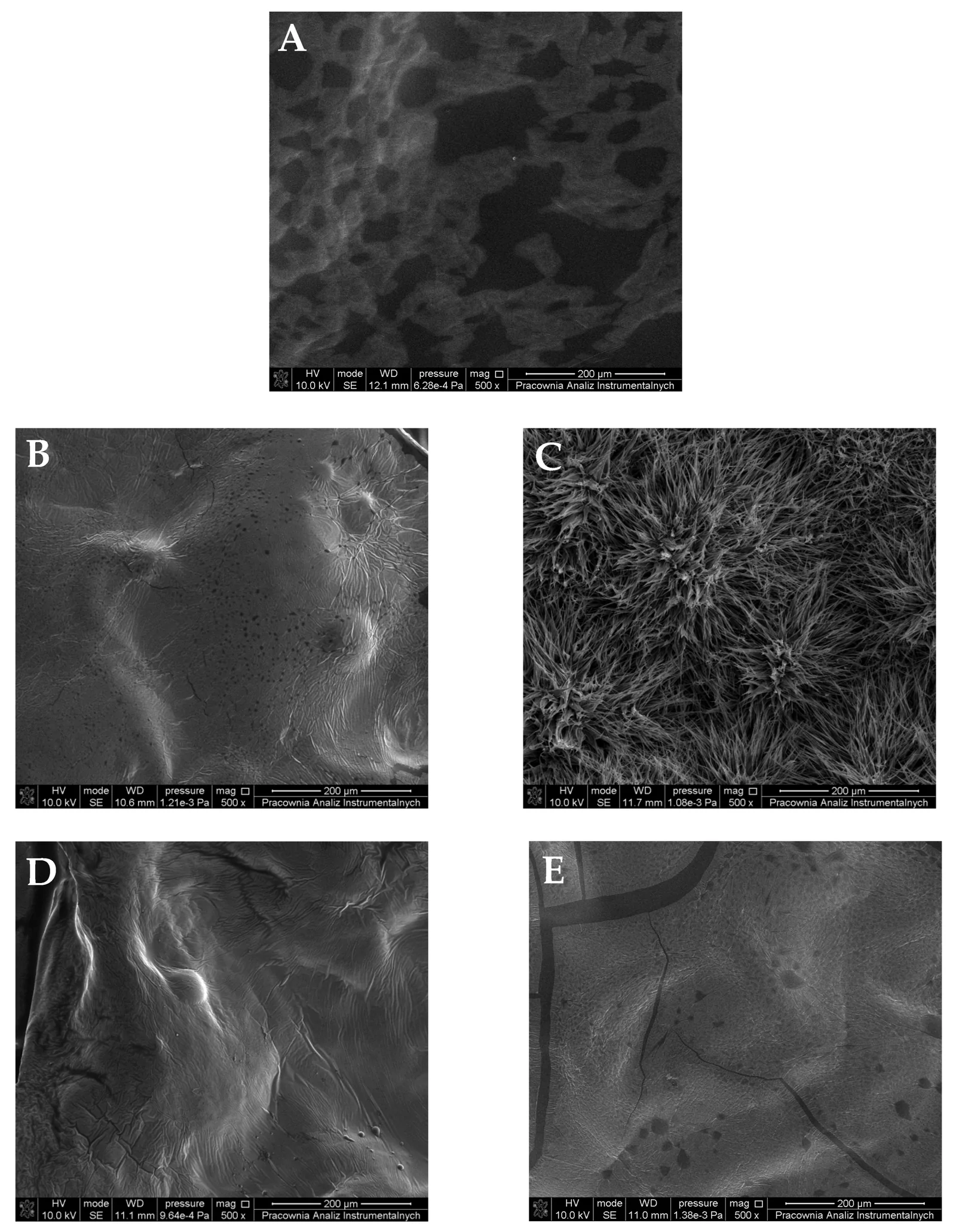
SEM images of lyophilized matrices: (**A**)—without extract and microspheres (CARR), (**B**)—with a hydroglycolic extract incorporated in gelatin microspheres (CARR/GEL (GE)), (**C**)—with a hydroglycolic extract in the entire volume of the matrix (CARR/GE), (**D**)—with an aqueous solution of dry extract incorporated in gelatin microspheres (CARR/GEL (AE)), (**E**)—with an aqueous solution of dry extract in the entire volume of the matrix (CARR/AE).

Figure 4 illustrates the surface morphology of the freeze-dried carrageenan-based hydrogel matrices observed by SEM. SEM images of the freeze-dried matrices revealed differences in surface morphology among the investigated formulations. The CARR matrix showed a relatively uniform surface with visible pores and cavities, whereas the extract-containing samples exhibited more heterogeneous and irregular surface structures. Differences in surface texture and pore appearance were observed among the individual formulations.

### 3.2. Mechanical Properties

The compressive moduli (E_c_) of different carrageenan-based hydrogels are presented in Table 2.

The compressive modulus (E_c_) of the investigated carrageenan-based hydrogels ranged from 14.10 ± 1.15 to 23.20 ± 1.46 kPa (Table 2). The lowest E_c_ value was recorded for the reference CARR hydrogel (14.10 ± 1.15 kPa), while the highest value was observed for CARR/GE (23.20 ± 1.46 kPa). Among the formulations containing the aqueous solution of the dry extract, CARR/GEL(AE) exhibited a higher compressive modulus (18.83 ± 2.58 kPa) than CARR/AE (14.90 ± 3.54 kPa). In contrast, CARR/GEL(GE) showed an E_c_ value (14.65 ± 0.64 kPa) comparable to that of the reference CARR matrix.

### 3.3. Swelling Properties

**Figure 5:**
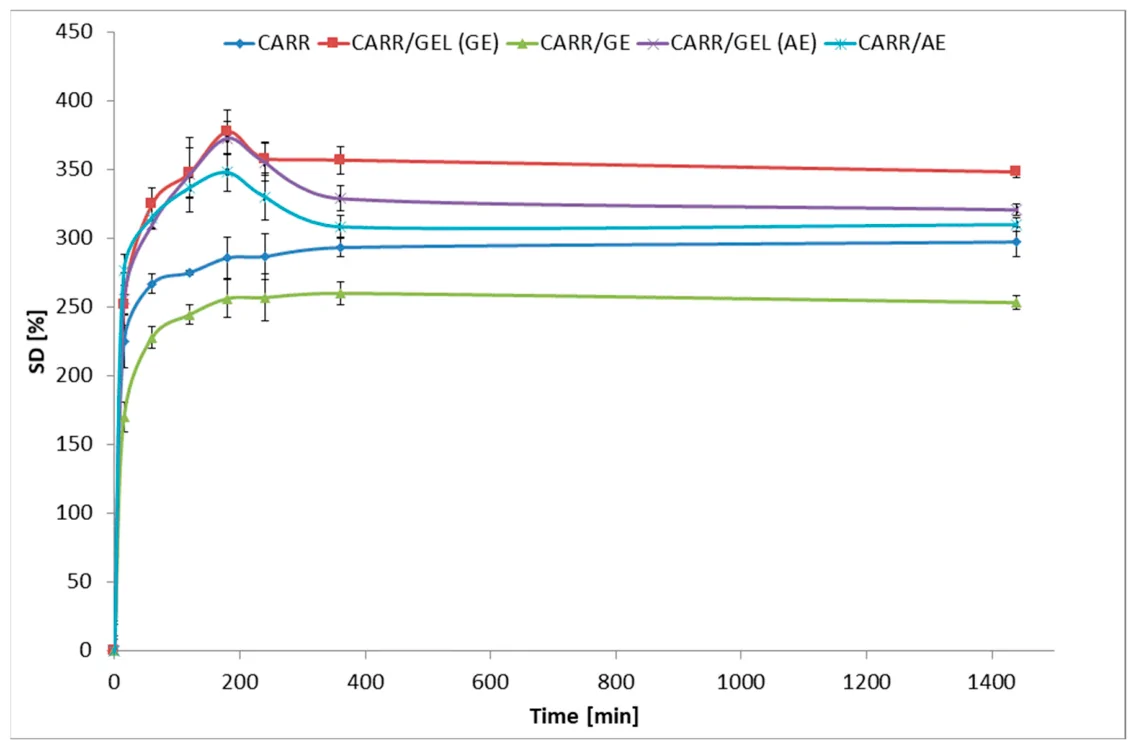
Swelling degree (SD) of carrageenan-based hydrogels as a function of immersion time.

The swelling properties of prepared carrageenan-based hydrogels as a function of time are shown in Figure 5.

The swelling behaviour of the carrageenan-based hydrogels over 24 h is presented in Figure 5. All investigated formulations exhibited a rapid increase in the degree of swelling during the initial stage of the experiment, followed by smaller changes at longer immersion times. CARR/GE consistently exhibited the lowest degree of swelling (~250% after 24 h), whereas CARR/GEL(GE) exhibited the highest values, reaching approximately 370% during the first hours of immersion and about 350% after 24 h. After 24 h, all hydrogels remained highly swollen, although differences among the formulations were still apparent.

### 3.4. Thermal Stability

**Figure 6:**
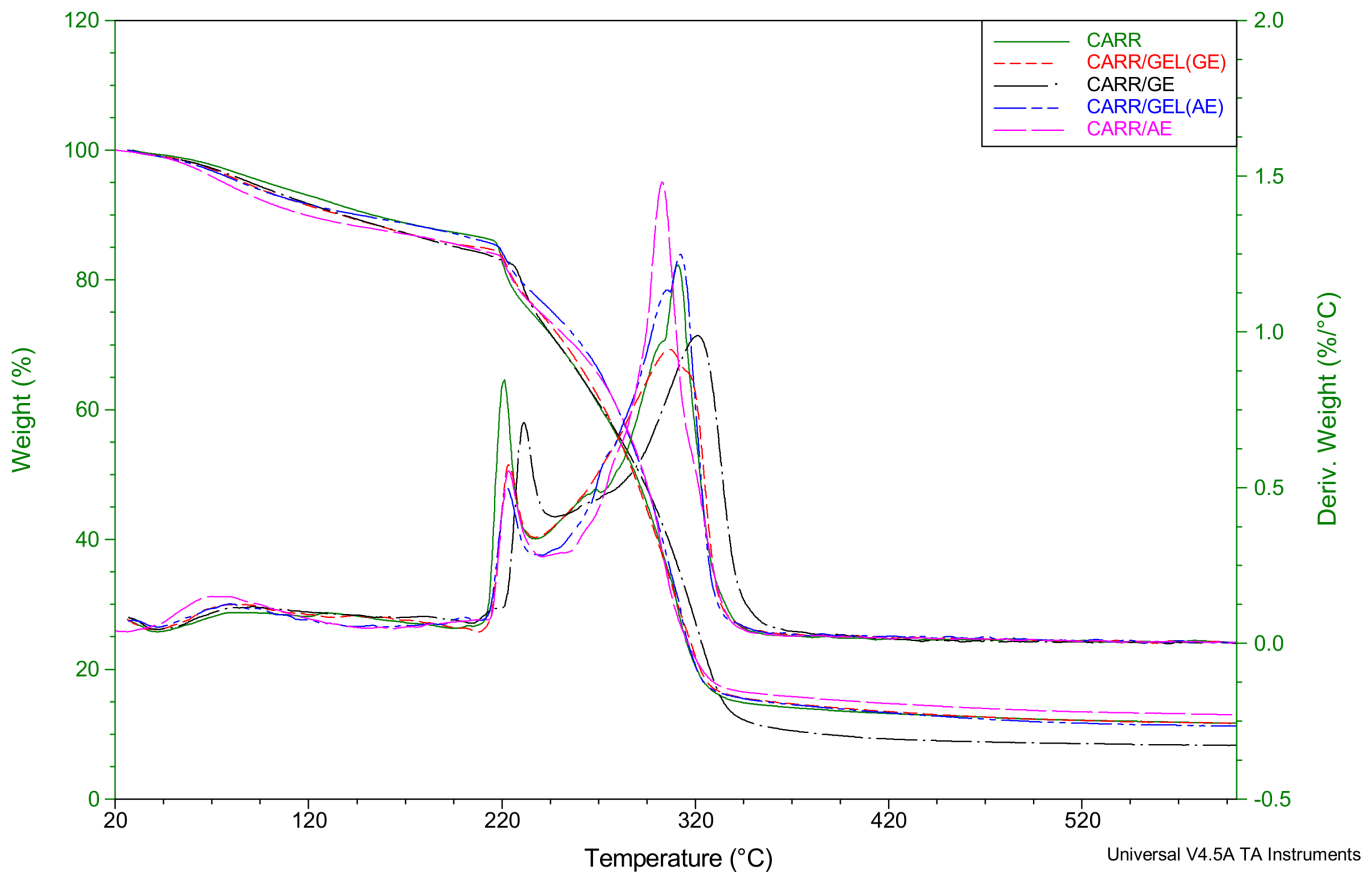
TG and DTG curves of carrageenan-based matrices.

The results of the thermal analysis are presented in Table 3 and Figure 6.

The thermal behaviour of the carrageenan-based matrices was evaluated using the TG and DTG curves (Figure 6), and the corresponding thermal parameters are summarized in Table 3. All investigated formulations exhibited three main stages of weight loss. The first stage was associated with a relatively small weight loss, ranging from 6.96% for CARR to 10.11% for CARR/AE. The second stage accounted for approximately 24.5–32.3% of the initial sample weight, with T_max_ values ranging from 209.0 to 231.3 °C. The third stage was characterized by the greatest weight loss, ranging from 49.07 to 54.86%, and T_max_ values between 302.9 and 333.9 °C. Among the investigated formulations, the reference CARR matrix exhibited the highest T_max_ in the third degradation stage (333.9 °C), whereas the lowest value was recorded for CARR/AE (302.9 °C).

### 3.5. Biophysical Skin Parameters

The biophysical skin parameters, such as skin barrier quality and skin colour, were examined using Courage + Khazaka probes. Table 4 summarizes the skin parameters of the volunteers enrolled in the study.

**Figure 7:**
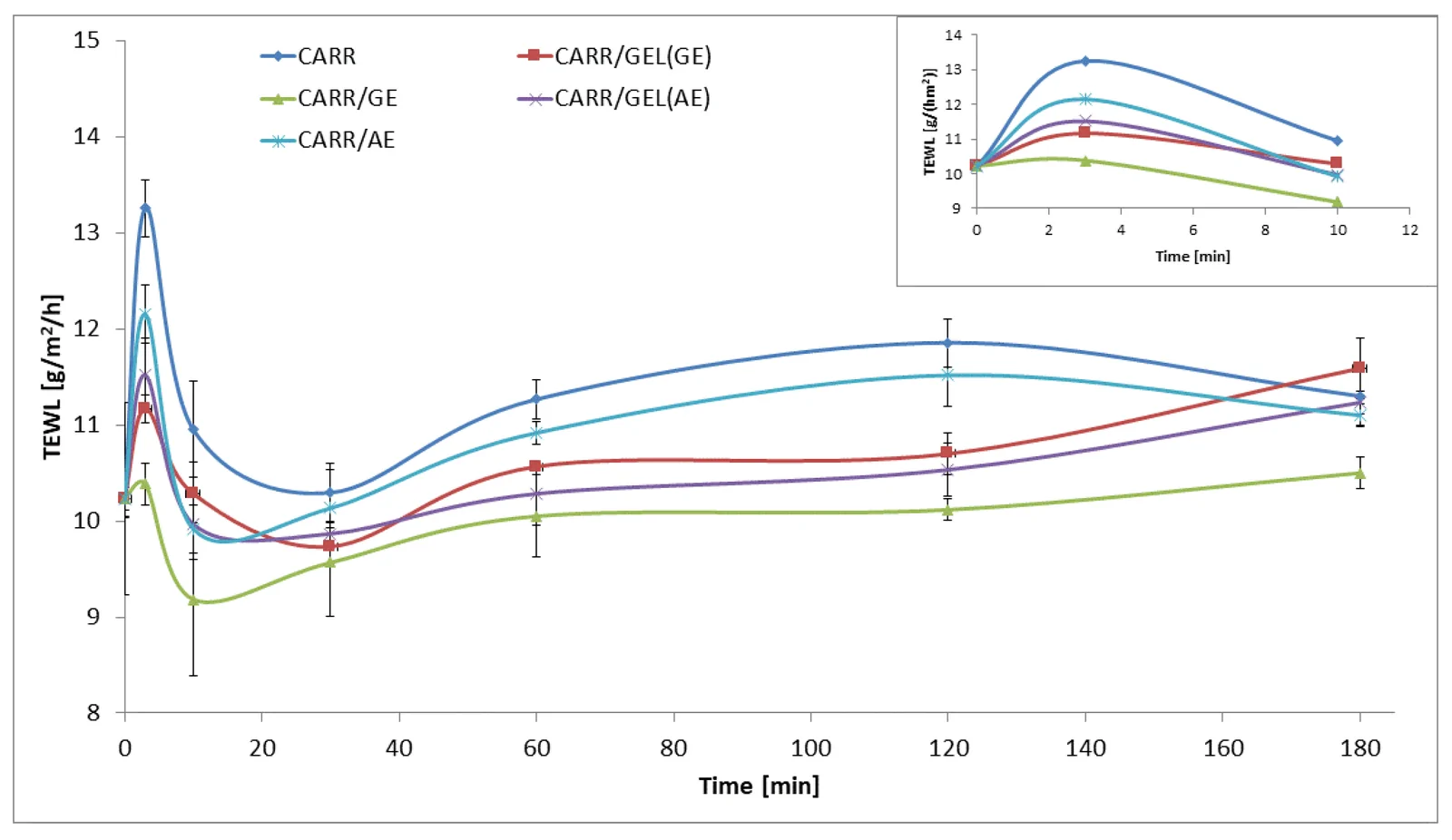
Tewametric measurements of skin before (control) and after topical application of the prepared hydrogels.

**Figure 8:**
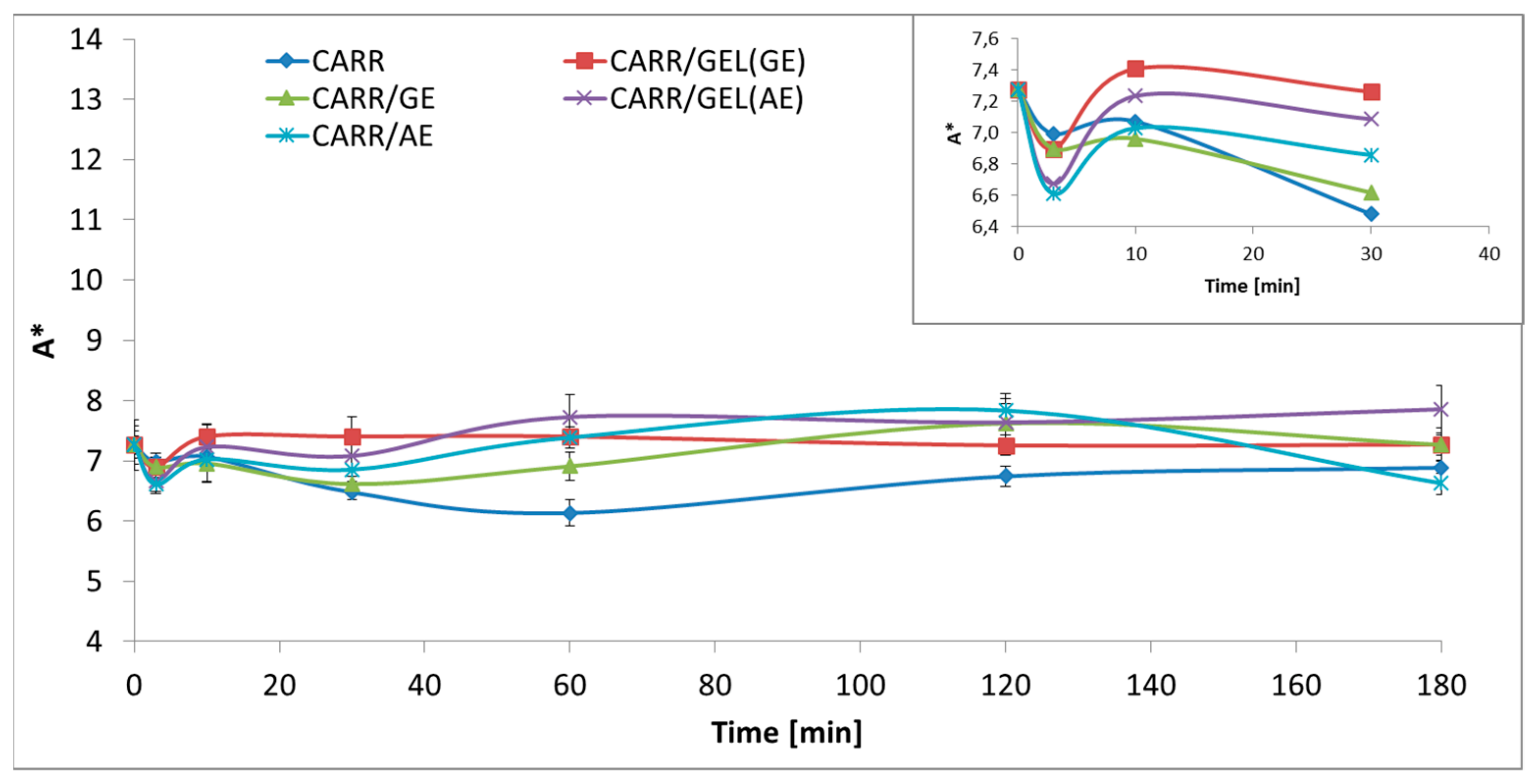
Colourimetric measurements of skin before (control) and after topical application of the prepared hydrogels.

The volunteers enrolled in the pilot study were relatively homogeneous in age and baseline skin characteristics. Baseline TEWL values were low, indicating a well-preserved epidermal barrier [32], while ITA values ranged from 55.3° to 62.0°, corresponding to very light skin pigmentation [33]. The Individual Typology Angle (ITA), calculated from the L* and b* coordinates of the CIELAB colour space, is commonly used to classify skin pigmentation, with higher ITA values corresponding to lighter skin tones. For the colourimetric assessment following hydrogel application, the analysis focused on the A* parameter, which represents the green–red axis of the CIELAB colour space and reflects changes in skin redness [33]. The results of these preliminary analyses following the application of carrageenan-based hydrogels are presented in Figure 7 and Figure 8. 

TEWL values showed relatively small variations following hydrogel application compared with the baseline values (Figure 7), with no pronounced increase observed for any of the tested formulations.

Measurements of the A* parameter revealed only minor changes following hydrogel application (Figure 8). The A* values remained relatively stable during the measurement period.

## 4. Discussion

The physicochemical properties of κ-carrageenan-based hydrogels varied with the type of *Calendula officinalis* preparation and the formulation strategy used for its incorporation. Direct addition of the extracts and their incorporation via gelatin microspheres resulted in distinct changes in hydrogel morphology, mechanical properties, swelling behaviour, and thermal characteristics. Both the extract and the particulate carrier may therefore contribute to the organization and macroscopic properties of the carrageenan network. It should be emphasized that two distinct crosslinking mechanisms were involved in the preparation of the systems investigated. The gelatin microspheres were chemically crosslinked using EDC/NHS, whereas the κ-carrageenan hydrogel matrix was formed by Ca^2+^-mediated ionic crosslinking. These mechanisms are well established and have been described previously in the literature [34,35].

Differences in the morphology of the freeze-dried matrices were also observed among the formulations. The reference CARR matrix exhibited a relatively porous and irregular structure, whereas incorporating the extracts, either directly or through gelatin microspheres, resulted in more heterogeneous morphologies and altered surface texture and pore appearance. The formation of κ-carrageenan hydrogels involves conformational organization of carrageenan chains into helical structures, followed by cation-mediated association and aggregation into a three-dimensional network. Thus, the carrageenan network investigated in the present study was stabilized by ionic interactions. This process is strongly dependent on ionic conditions and can also be affected by other macromolecular constituents present in the formulation [3,7]. One possible explanation is that the introduction of plant-extract constituents or an additional gelatin phase may modify local polymer-polymer and polymer-solvent interactions and, consequently, influence the organization of the network formed during gelation. However, the present stereomicroscopic and SEM observations provide morphological evidence only and do not directly demonstrate these molecular interactions.

Previous studies on composite hydrogels containing carrageenan have similarly demonstrated that combining carrageenan with other polymers, such as gelatin, chitosan, alginate, starch, or poly(vinyl alcohol), can alter pore architecture and the overall morphology of the resulting matrices [10,11,12]. Plant-derived extracts have also been successfully incorporated into carrageenan-containing hydrogel systems. For example, Ditta et al. developed agarose/κ-carrageenan hydrogel films, including a formulation loaded with an aqueous extract of Cryphaea heteromalla, and characterized their morphology by SEM, as well as their mechanical and swelling properties [36]. However, direct evidence linking the incorporation of plant extracts to specific morphological changes in carrageenan networks remains limited. Therefore, the morphological differences observed in the present study cannot be attributed to a specific interaction or structural mechanism.

The reference CARR matrix exhibited a compressive modulus of 14.10 ± 1.15 kPa, whereas direct incorporation of the hydroglycolic extract increased E_c_ to 23.20 ± 1.46 kPa, corresponding to an approximately 65% increase. Since κ-carrageenan contains sulfate groups, its network organization is strongly influenced by electrostatic interactions and ionic associations, particularly those involving counterions [3,7]. The increased compressive modulus of CARR/GE represents a directly measured mechanical effect. One possible explanation is that the hydroglycolic extract preparation altered intermolecular interactions and/or polymer-chain mobility within the carrageenan-based matrix, resulting in a more mechanically constrained network. Such effects may arise from the combined contribution of extract constituents and the hydroglycolic vehicle. However, this interpretation remains hypothetical, as the detailed phytochemical composition of the commercial preparation was not determined and the molecular interactions within the hydrogel network were not directly investigated. Therefore, the contribution of individual components cannot be established from the present data.

The relationship between supramolecular organization and mechanical behaviour has also been demonstrated for carrageenan-based multicomponent systems. Makarova et al. showed that the formation of a common supramolecular structure in κ-carrageenan-gelatin gels, involving increased intermolecular protein-polysaccharide contacts, was associated with changes in the mechanical and rheological properties of the composite system [37]. Interactions between κ-carrageenan and other formulation components can therefore affect the mechanical response of the resulting network.

The pronounced increase in stiffness observed for CARR/GE was not maintained when the hydroglycolic extract was incorporated into gelatin microspheres. CARR/GEL(GE) exhibited an E_c_ of 14.65 ± 0.64 kPa, which was close to that of the reference CARR matrix. Localization of the extract within the microspheres may have limited direct interactions between some extract constituents and the continuous carrageenan phase. At the same time, incorporation of gelatin microspheres introduced a dispersed polymeric phase and additional particle-matrix interfaces, which may also contribute to the mechanical behaviour of the composite hydrogel. The gelatin microspheres were chemically crosslinked using the EDC/NHS system, which is commonly used to promote covalent amide bond formation between carboxyl and amino groups in gelatin-based systems [34]. Similar effects of incorporating a dispersed particulate phase into carrageenan-based hydrogel matrices have been reported by Pettinelli et al., who showed that PHBV microparticles embedded in a physically crosslinked κ-carrageenan/locust bean gum hydrogel altered its morphology and rheological properties [38]. However, the effect of the microparticles on the rheological properties depended on the measurement conditions, indicating that the incorporation of a particulate phase does not necessarily result in a uniform reinforcing effect.

A different response was observed for the aqueous solution of the dry extract. Direct incorporation resulted in an E_c_ of 14.90 ± 3.54 kPa, which remained close to that of CARR, whereas CARR/GEL(AE) exhibited a higher modulus of 18.83 ± 2.58 kPa. Thus, the mechanical response associated with microsphere-mediated incorporation differed between the two extract types. These results suggest that the mechanical behaviour of the developed hydrogels was determined by the combined effects of the continuous carrageenan network, the dispersed gelatin phase, and the composition and localization of the extract, rather than by the presence of gelatin microspheres alone.

The swelling results provide complementary information on the organization of the investigated hydrogel networks. All formulations displayed rapid water uptake during the initial immersion period, followed by smaller changes at longer times. Such behaviour is consistent with the highly hydrophilic nature of carrageenan-based hydrogel networks. Carrageenan is a hydrophilic sulfated polysaccharide, while other hydrophilic components present in the investigated matrices, including collagen, gelatin, glycerin, sorbitol, and propylene glycol, may additionally contribute to water uptake. High water absorption is a well-established characteristic of carrageenan-based hydrogels [3,4,9].

CARR/GE exhibited both the highest compressive modulus and the lowest degree of swelling. A more constrained network with reduced polymer-chain mobility would be expected to resist deformation more effectively while limiting network expansion upon contact with water. Thus, the combination of high E_c_ and reduced SD observed for CARR/GE is consistent with a more mechanically constrained network. A relationship between network organization, swelling, and mechanical properties of κ-carrageenan hydrogels has also been demonstrated by Wurm et al. [39]. The authors showed that extensive pre-drying led to irreversible network densification accompanied by reduced swelling. In contrast, ion-induced restructuring of the κ-carrageenan network during swelling was associated with an increase in the storage modulus. These observations demonstrate the relationship between the ability of the carrageenan network to expand and its mechanical organization. Similarly, in our previous study, variations in sorbitol and glycerin content were associated with changes in both the swelling and mechanical properties of κ-carrageenan hydrogels [9].

A different relationship between swelling and mechanical properties was observed for CARR/GEL(GE). This formulation exhibited a compressive modulus close to that of CARR but showed the highest swelling degree, particularly during the first hours of immersion. Incorporation of the extract-loaded gelatin microspheres was associated with greater uptake of the aqueous medium without a proportional increase in bulk stiffness. High water-holding capacity has previously been reported for gelatin-carrageenan hydrogels. Alcantara et al. found that hydrogels prepared at different gelatin-to-carrageenan ratios retained approximately 80–90% of water [40]. The authors also demonstrated that the gelatin-to-carrageenan ratio influenced the structural and rheological properties of the resulting networks. Although water-holding capacity and degree of swelling are distinct parameters, these results are consistent with the high hydration capacity of gelatin-carrageenan networks. In the present microsphere-containing hydrogels, the presence of both the continuous carrageenan network and the dispersed gelatin phase may therefore contribute to the hydration behaviour of the material. This provides a plausible explanation for the high SD of CARR/GEL(GE) despite its compressive modulus remaining close to that of the reference hydrogel.

The swelling and mechanical results indicate that a simple inverse relationship between swelling and stiffness does not hold for all investigated formulations. The microsphere-containing formulations differ from a simple homogeneous carrageenan network, as their properties are influenced not only by the organization of the carrageenan matrix, but also by the presence of gelatin microspheres and the localization of the extract. Therefore, the observed differences in swelling and mechanical behaviour likely result from the combined contribution of these factors. Whereas CARR/GE showed higher stiffness accompanied by lower swelling, the microsphere-containing systems represent heterogeneous composite networks in which the continuous carrageenan phase, the dispersed gelatin particles, and the localization of the extract may contribute differently to swelling and mechanical resistance. In the present systems, microsphere-mediated incorporation was associated with changes in hydration properties that were not accompanied by proportional changes in bulk mechanical behaviour. From the perspective of topical application, this combination of properties may be advantageous, as sufficient mechanical integrity is important for material handling and maintaining contact with the skin, while water uptake helps maintain the hydrated character of the hydrogel.

Thermogravimetric analysis provided complementary information on the thermal behaviour of the investigated matrices. All formulations exhibited three major mass-loss stages, indicating a broadly similar thermal degradation pattern despite differences in their composition. A relatively small mass loss characterized the first stage and is primarily attributable to the removal of free and bound water and other low-molecular-weight volatile components. Similar low-temperature mass-loss events have been reported for κ-carrageenan-based materials and attributed predominantly to water evaporation associated with the hydrophilic character of the polysaccharide [41]. The corresponding T_max_ shifted from 42.2 °C for CARR and 42.8 °C for CARR/GE to 82.2 and 79.5 °C for CARR/GEL(GE) and CARR/GEL(AE), respectively. The shift in T_max_ may reflect differences in the state and strength of water binding in the microsphere-containing matrices. A similar shift in moisture-related DTG events towards higher temperatures was reported for chemically crosslinked gelatin/κ-carrageenan hydrogels and was attributed to tighter binding of water within the crosslinked network [40]. The presence of crosslinked gelatin microspheres may therefore have contributed to differences in water binding within the investigated matrices. Nevertheless, since different water populations were not directly characterized in the present study, this mechanism should be regarded as a plausible explanation rather than a definitive assignment.

The second and third mass-loss stages involved substantially greater mass losses and most likely represent overlapping degradation processes of the organic components of the matrices. Multistep thermal degradation has also been reported for κ-carrageenan-based materials, with moisture loss occurring at lower temperatures and degradation of the polymeric components becoming predominant at higher temperatures [41,42]. However, the matrices investigated in the present study contained κ-carrageenan, collagen, sorbitol, glycerin, propylene glycol, constituents of C. officinalis extract, and, in selected formulations, gelatin. Consequently, assignment of individual DTG maxima to a single component would not be justified without thermal analysis of the individual constituents under identical experimental conditions.

Nevertheless, comparison of the third degradation stage revealed a clear formulation-dependent effect. The reference CARR matrix exhibited the highest T_max_ (333.9 °C), whereas lower values ranging from 302.9 to 321.6 °C were recorded for the extract-containing formulations. Thus, incorporation of the extracts, either directly or through gelatin microspheres, did not increase the high-temperature thermal stability of the reference matrix. The influence of plant-derived components on the thermal behaviour of κ-carrageenan materials has also been demonstrated by Bhatia et al. [41], who showed that incorporation of grapefruit essential oil altered the TGA/DTG profiles and increased the thermal resistance of κ-carrageenan films. This effect differed from that observed in the present study, where the extract-containing formulations exhibited lower T_max_ values for the third degradation stage than the reference CARR matrix. These findings indicate that the effect of plant-derived additives on the thermal behaviour of carrageenan-based materials may depend on their chemical composition and interactions with the polymer matrix.

The thermal and mechanical results did not follow the same trend. CARR/GE exhibited the highest compressive modulus but a lower third-stage T_max_ (321.6 °C) than the reference CARR matrix (333.9 °C). Thus, increased mechanical stiffness did not correspond to increased thermal stability. This distinction is reasonable because the two techniques probe different material characteristics: the compressive modulus reflects the resistance of the hydrated network to deformation under ambient conditions, whereas TGA monitors mass loss associated with thermally induced physicochemical and chemical transformations. Interactions contributing to a more mechanically constrained network under ambient conditions may not necessarily increase its resistance to thermal degradation. Consequently, mechanical stiffness and thermal stability should be considered complementary rather than directly correlated properties of these multicomponent hydrogels.

TEWL is widely used to assess epidermal barrier function by monitoring water flux through the skin [32]. In the present pilot study, no pronounced persistent increase in TEWL was observed following application of the hydrogels. This observation suggests that short-term contact with the formulations did not produce an evident increase in epidermal water loss under the experimental conditions. However, TEWL values obtained shortly after exposure to a highly hydrated material should be interpreted cautiously, as surface hydration and transient occlusive effects may influence water evaporation.

Colourimetric measurements provided an additional parameter for monitoring the skin response. The analysis focused on the A* coordinate of the CIELAB colour space, which represents the green–red axis and therefore reflects changes in skin redness [33]. Only minor variations in A* were observed after hydrogel application, with no clear, persistent increase during the measurement period. Together with the TEWL results, the relatively stable A* values indicate that no obvious acute changes in skin redness or epidermal water loss were observed following a single 20 min application. However, considering the very small number of volunteers, these observations should be regarded exclusively as preliminary.

The skin-related properties of C. officinalis-containing formulations have previously been investigated in clinical and experimental studies. Recently, Šušak Crnčević et al. evaluated an emollient cream containing 1% supercritical CO_2_ extract of C. officinalis in comparison with the corresponding placebo emollient in an experimentally induced model of irritant contact dermatitis. The formulation containing C. officinalis extract resulted in significantly greater skin hydration than the untreated control and placebo at selected time points and a significantly lower TEWL value than the untreated control on day 3. No significant differences in erythema were observed between the treatment groups [43]. Importantly, the experimental conditions of that study differed substantially from those of the present pilot evaluation. In the study by Šušak Crnčević et al., the skin barrier was deliberately impaired by exposure to sodium lauryl sulfate, whereas the hydrogels investigated in the present study were applied to intact skin. Therefore, the present results cannot be interpreted as evidence of an anti-inflammatory or barrier-restoring effect of C. officinalis extract. They indicate only that short-term application of the developed hydrogels was not associated with pronounced persistent changes in TEWL or skin redness under the experimental conditions used.

The skin measurements should be regarded as a preliminary proof-of-concept evaluation rather than an efficacy study. The small number of volunteers (n = 3) precludes robust statistical assessment and generalization of the observed responses. Nevertheless, the combined evaluation of TEWL and A* provides useful initial information on the short-term skin response to the formulations and supports further investigation in a larger study population.

The properties of the developed κ-carrageenan-based hydrogels differed depending on the formulation strategy used for extract incorporation. Direct incorporation of the hydroglycolic extract produced the most pronounced changes, resulting in the highest compressive modulus and the lowest degree of swelling. In contrast, incorporation of the same extract through gelatin microspheres resulted in mechanical properties close to those of the reference hydrogel and greater water uptake. A different response was observed for the aqueous solution of the dry extract, indicating that the effects associated with microsphere-mediated incorporation also depended on the type of encapsulated extract. The morphological, mechanical, swelling, and thermal results are consistent with the gelatin microspheres constituting a dispersed polymeric phase within the carrageenan matrix, while encapsulation may also have altered the extent of direct contact between extract constituents and the continuous carrageenan phase. The results further demonstrate that changes in mechanical properties and water uptake were not directly proportional across the investigated formulations. This was particularly evident for CARR/GEL(GE), which exhibited a compressive modulus close to that of the reference hydrogel while showing substantially greater swelling. Thus, direct and microsphere-mediated incorporation of plant extracts represent different approaches to modifying the physicochemical properties of carrageenan-based hydrogel systems.

It should be emphasized that the microsphere-containing formulations differed from the directly loaded formulations not only in the localization of the extract but also in the presence of an additional gelatin phase. Therefore, the effects of extract encapsulation cannot be separated from the physicochemical contribution of the gelatin microspheres themselves. In the absence of a control formulation containing unloaded gelatin microspheres, the observed differences should be interpreted as the combined effect of the dispersed gelatin phase and the altered localization of the extract within the hydrogel matrix.

Several limitations should nevertheless be acknowledged. The commercial C. officinalis extracts were not subjected to detailed phytochemical characterization. The release of extract constituents from either the carrageenan matrix or the gelatin microspheres was not investigated. Therefore, the present study demonstrates incorporation of the commercial extract preparations and physicochemical characterization of the resulting hydrogel systems, but does not provide evidence of controlled or effective delivery of *Calendula officinalis* constituents. These limitations restrict interpretation of the relationships between extract composition, formulation strategy, and the resulting hydrogel properties. Future studies should therefore include phytochemical characterization of the extracts, release studies, and skin evaluation in a larger study population.

## 5. Conclusions

The present study demonstrated that *Calendula officinalis* flower extracts can be incorporated into κ-carrageenan-based hydrogels either directly or through gelatin microspheres, with the resulting formulations showing distinct physicochemical properties. Differences among the formulations were observed in morphology, mechanical behaviour, swelling, and thermal characteristics. The formulation containing directly incorporated hydroglycolic extract showed the highest compressive modulus and the lowest degree of swelling, consistent with a more mechanically constrained hydrogel network. In contrast, the corresponding microsphere-containing formulation showed mechanical properties close to those of the reference hydrogel while exhibiting greater water uptake. The observed differences between the directly incorporated and microsphere-containing formulations reflected differences between the overall formulation strategies, including the presence of gelatin microspheres, the localization of the extract within the hydrogel system, and potentially different extract loading. Preliminary skin measurements showed no pronounced persistent increase in TEWL or marked changes in the A* parameter following short-term hydrogel application. Further studies should include phytochemical characterization of the extracts, evaluation of extract release from the hydrogel matrices, and skin assessment in a larger study population. Release studies will be required to determine whether these systems can provide controlled or effective delivery of *Calendula officinalis* constituents.

## Figures and Tables

**Table 1:** Composition of the κ-carrageenan-based hydrogel formulations.

Component	Composition of Hydrogels [%]				
	CARR	CARR/GEL (GE)	CARR/GE	CARR/GEL (AE)	CARR/AE
Water	75.4	75.4		75.4	-
Hydroglycolic *C. officinalis* extract solution (0.1% *w/v*, dry-matter equivalent)	-	-	75.4	-	-
Aqueous solution of dry *C. officinalis* extract (0.1% *w/v*)	-	-	-	-	75.4
Sorbitol (60%)	20	20	20	20	20
Carrageenan	2	2	2	2	2
Glycerin	1.5	1.5	1.5	1.5	1.5
Propylene glycol	1	1	1	1	1
Collagen	0.1	0.1	0.1	0.1	0.1
Gelatin microspheres	-	1	-	1	-

**Table 2:** Compressive modulus [E_c_] of different carrageenan-based hydrogels (*n* = 5; * significantly different from CARR (*p* < 0.05)).

	E_c_ [kPa]
CARR	14.10 ± 1.15
CARR/GEL(GE)	14.65 ± 0.64
CARR/GE	23.20 ± 1.46 *
CARR/GEL(AE)	18.83 ± 2.58
CARR/AE	14.90 ± 3.54

**Table 3:** Thermal parameters of carrageenan-based hydrogels.

	Stage 1		Stage 2		Stage 3	
	Weight Loss (%)	T_max_ (°C)	Weight Loss (%)	T_max_ (°C)	Weight Loss (%)	T_max_ (°C)
CARR	6.96	42.2	32.28	221.2	49.07	333.9
CARR/GEL(GE)	8.56	82.2	29.59	209.0	50.16	307.1
CARR/GE	8.27	42.8	30.73	231.3	52.73	321.6
CARR/GEL(AE)	8.36	79.5	25.51	210.4	54.86	312.5
CARR/AE	10.11	68.7	24.53	223.4	52.33	302.9

**Table 4:** Biophysical skin parameters and age of the volunteers enrolled in the study.

Skin Parameters and Age of the Study Participants				
Subject Number	1	2	3	Average
TEWL [g/m^2^/h]	8.4	11.9	10.5	10.2
A*	8.1	6.0	7.7	7.3
ITA [°]	55.3	62.0	59.3	58.9
Age	22.0	23.0	22.0	22.3

## Data Availability

The data that support the findings of this study are available from the corresponding author upon reasonable request.

## Abbreviations

The following abbreviations are used in this manuscript:

AE	Aqueous solution of dry *Calendula officinalis* extract
CARR	κ-Carrageenan-based hydrogel
CARR/AE	κ-Carrageenan-based hydrogel with directly incorporated aqueous solution of dry *Calendula officinalis* extract
CARR/GE	κ-Carrageenan-based hydrogel with directly incorporated hydroglycolic *Calendula officinalis* extract solution
CARR/GEL(AE)	κ-Carrageenan-based hydrogel containing gelatin microspheres loaded with an aqueous solution of dry *Calendula officinalis* extract
CARR/GEL(GE)	κ-Carrageenan-based hydrogel containing gelatin microspheres loaded with hydroglycolic *Calendula officinalis* extract solution
CIELAB	Commission Internationale de l’Éclairage L*a*b* colour space
DTG	Derivative thermogravimetry
E_c_	Compressive modulus
EDC	1-Ethyl-3-(3-dimethylaminopropyl)carbodiimide
GE	hydroglycolic *Calendula officinalis* extract solution
ITA	Individual Typology Angle
NHS	N-Hydroxysuccinimide
SD	Swelling degree
SEM	Scanning electron microscopy
TEWL	Transepidermal water loss
TG	Thermogravimetry
T_max_	Temperature of maximum mass-loss rate
